# Pathogenesis and Management of Acute Kidney Injury in Patients with Nephrotic Syndrome Due to Primary Glomerulopathies

**DOI:** 10.3390/medicina55070365

**Published:** 2019-07-11

**Authors:** Sophia Lionaki, George Liapis, John N. Boletis

**Affiliations:** 1 Nephrology Department & Transplantation Unit, Laiko Hospital, Faculty of Medicine, National & Kapodistrian University of Athens, 11527 Athens, Greece; 2 Department of Pathology, Laiko Hospital, Faculty of Medicine, National & Kapodistrian University of Athens, 11527 Athens, Greece

**Keywords:** glomerular diseases, acute kidney injury, nephrotic syndrome

## Abstract

Acute kidney injury in the context of nephrotic syndrome is a serious and alarming clinical problem. Largely, acute kidney injury is a relatively frequent complication among patients with comorbidities while it has been independently associated with an increased risk of adverse outcomes, including death and chronic kidney disease. Nephrotic syndrome, without hematuria or with minimal hematuria, includes a list of certain glomerulopathies; minimal change disease, focal segmental glomerulosclerosis and membranous nephropathy. In the light of primary nephrotic syndrome, pathophysiology of acute kidney injury is differentiated by the nature of the primary disease and the severity of the nephrotic state. This review aims to explore the clinical circumstances and pathogenetic mechanisms of acute kidney injury in patients with nephrotic syndrome due to primary glomerulopathies, focusing on newer perceptions regarding the pathogenesis and management of this complicated condition, for the prompt recognition and timely initiation of appropriate treatment in order to restore renal function to its baseline level. Prompt recognition of the precise cause of acute kidney injury is crucial for renal recovery. Clinical characteristics, laboratory and serological findings along with histopathological findings, if required, will reveal the implicated pathway leading to individualized approach and management.

## 1. Introduction

Acute kidney injury (AKI), in the context of nephrotic syndrome (NS), is a serious and alarming clinical problem. Largely, AKI is frequent among patients with comorbidities, while it has been correlated with an increased frequency of adverse outcomes, including death [1,2,3], and chronic renal failure [4]. Specifically, death rates have been reported to increase with AKI severity from 15.9% in stage I, to 49.3% in dialysis-dependent patients [3]. Of those who become dialysis-dependent during hospitalization but come off dialysis and are discharged subsequently, 5%–20% will remain dialysis-dependent [4]. The risk of developing end stage kidney disease has been reported to increase 2-fold by one year for patients who recover from AKI within 10 days [5], while the risk further increases with the number of AKI episodes [6,7,8]. Patients with glomerular diseases are prone to develop AKI, either as a result of the aggressive form of the disease, or in relation with factors surrounding the pathophysiology of the glomerular lesion and its treatment. Nephrotic syndrome (without hematuria or with minimal hematuria) includes a list of many diseases; with respect to primary glomerulopathies, the commonest are minimal change disease, focal segmental glomerulosclerosis and membranous nephropathy. Acute kidney injury is classified by the main pathophysiologic mechanism involved in prerenal, intrinsic renal and postrenal causes. Theoretically, any of these scenarios may occur in patients with NS due to primary glomerulopathy, although the prerenal and intrinsic ones are the most frequent seen in this setting.

This review aims to explore the clinical circumstances and pathogenetic mechanisms of AKI in patients with NS, focusing on newer perceptions regarding the pathogenesis and management of this complicated condition, for the prompt recognition and timely initiation of appropriate treatment in order to restore renal function to its baseline level.

## 2. Definitions and Epidemiology of AKI

Acute kidney injury (AKI) is defined by an unexpected decrease in the glomerular filtration rate (GFR), leading to the retention of nitrogenous waste products, as well as the dysregulation of extracellular volume and electrolytes. According to the Kidney Disease Improving Global Outcomes (KDIGO) [9] definition, AKI is characterized by an increase in serum creatinine by ≥0.3 mg/dL within 48 h or to ≥1.5 times baseline, which is known or presumed to have occurred within the past seven days, or urine volume <0.5 mL/kg/h for six hours [9]. Correction of volume depletion and exclusion of obstructive uropathy by ultrasonography are typically preceded. Depending on the time and degree of creatinine increase and urine output volume, AKI is staged in three grades [9]. However, this criteria system does not distinguish between the multiple etiologies that cause AKI while the criterion which is referring to, the urine volume, is controversial as it is not based on robust evidence [10,11,12,13,14,15,16,17,18,19,20].

The precise frequency of AKI in the general population is not known, probably due to differences in the definitions and cohorts of patients enrolled across studies. A meta-analysis of 143 studies, in more than 3.5 million hospitalized patients, applying the KDIGO definitions, found that 22% of the patients experience this complication [3], increasing to 57.3% for the intensive care units [21]. The incidence of AKI has been shown to increase in recent years [22] with standard risk factors including pre-existing kidney disease, diabetic nephropathy, volume depletion, effective arterial volume depletion.

## 3. Frequency of AKI in Patients with NS

A modest reduction of GFR of about 30% is frequent among patients with NS, as shown in a series of 89 adults with biopsy-proven minimal change disease. In this series, diminished renal function was recorded in 22.5% of patients, typically aged over 60 years [23]. Similar findings have been found in other studies in adults and children [24,25,26,27]. Studies in children with NS have shown a close correlation of GFR alteration with both the reduced filtration fraction and the decreased serum albumin concentration that are relative to the severity of the nephrotic state. However, the frequency of AKI across patients with NS varies depending on the histopathological type and surrounding factors related to the patient and the management of NS itself. Smith et al. studied a series of cases with severe AKI which occurred in the clinical setting of idiopathic NS and found that, in 60% of them, certain abnormalities in the tubule-interstitial compartment were suggestive of acute tubular necrosis [28]. In children, hospitalized with NS, an incidence rate of 8.5% AKI has been reported [29,30]. More recently, there have been reported rates of AKI of 58.6% for 336 children and 50.9% for 615 hospitalizations [30]. After adjustments, factors such as infection and nephrotoxic medication exposure, including duration and intensity of exposure, were shown to be significantly associated with AKI in those patients. In adults, AKI rates have been reported to occur in 25%–35% of patients with minimal change disease, which in many occasions occur as part of the initial clinical presentation [28,31,32,33,34]. Adults with minimal change disease, who develop AKI, are more likely than those who do not develop AKI to be older, male, hypertensive, and to have more severe NS [35,36]. Waldman et al. [31], reported that AKI occurred in 25% of patients. In 70% of them, it was concurrent with the initial diagnosis of minimal change disease, while one third of them followed a disease relapse. Likewise, a study of 125 adults and adolescents from the Netherlands recorded AKI in 40% of them [36]. Patients with AKI in this context usually present with significant proteinuria, hypoalbuminemia, and edema. Acute kidney injury, in this context, occurred a few months after the NS diagnosis and the majority of patients had hypertension and massive edema at presentation [28]. Remarkably, most patients were also oliguric, and nearly 20% of them required dialysis or died from intercurrent complications. In general, GFR has been reported to be well-maintained during the follow-up, although serum creatinine was higher in those who had presented with AKI [34]. There are less reports from patients with membranous nephropathy [37] or focal segmental glomerulosclerosis [38], probably due to the lower frequency of this complication among these patients. Notably, among 235 elderly patients, in whom the most frequent indication for renal biopsy was AKI (46%), 9.4% of them presented with NS and AKI [37]. The most frequent diagnosis was minimal change disease, which was found in one-quarter of these patients.

## 4. Pathophysiology of NS

The historic definition of nephrotic syndrome requires the presence of triad proteinuria, typically 3.5 g per day or more, lipiduria and hyperlipidemia, and edema. A proportion of patients may also have microscopic hematuria, although typically the urine sediment is bland. The main abnormality in NS is the loss of protein in urine. Inability to reabsorb the filtered protein may result in proteinuria, i.e., 0.5–2.5 g/day, in patients with a normal GFR [39]. If proteinuria is more than 2–2.5 g/day it implies that at least part of the lost protein is due to increased glomerular permeability [39]. The type of urinary protein may provide some information regarding the stage of the glomerular lesion. For instance, highly selective proteinuria (only very small molecules are filtered) is associated with better histopathological findings. As a result of the significant protein excretion, comes hypoalbuminemia [40]. In response to the low serum albumin level, the liver increases not only albumin formation but also lipid production. Thus, low density lipoprotein cholesterol, low-density lipoproteins and apolipoproteins B, CII, and CIII, are found to be increased in NS [41,42,43,44,45], primarily as a consequence of altered colloid oncotic pressure and triglycerides being over-produced [43,44,45]. Edema formation in patients with NS is explained by the “underfill hypothesis” in which the kidney is retaining sodium and water in order to fix intravascular hypovolemia, a condition which ends up in the development of edema, due to the forces of Starling [46,47,48]. Conversely, the “overfill hypothesis” pertains to renal sodium retention, which occurs as a primary phenomenon which increases blood volume [49,50]. However, the primary defect, as noted by Rostoker et al., is the abnormal glomerular permeability, a problem which is often remitted after treatment with glucocorticoids [51].

Hypercoagulability is another major complication of NS, which is occurs secondarily to impaired coagulation and fibrinolysis [44]. Plasma viscosity increases in conditions with reduced blood flow, increased interstitial pressure and endothelial injury [52]. Loss of antithrombin III is also particularly important since patients who lack this factor have a 50%–70% risk of experiencing thromboembolic events [53]. Citak et al. [53] showed that pediatric nephrotic patients who experienced thrombotic events had very decreased antithrombin III and increased fibrinogen when compared with those without any thrombosis. Moreover, factors XI, XII, and plasminogen [52,54,55,56] are lost in urine in the cases with massive proteinuria. Yet, procoagulant factors, including factors V, VII, VIII, and the von Willebrand factor, have been reported to increase due to hypoalbuminemia, because they are normally bound to albumin [52,55]. Additional problems in nephrotic patients include increased susceptibility to infection and atherosclerosis, malnutrition, hormone dysregulation and a vitamins and metals deficiency [42].

## 5. Pathophysiology of AKI in Patients with NS

### 5.1. Acute Tubular Necrosis

**Figure 1:**
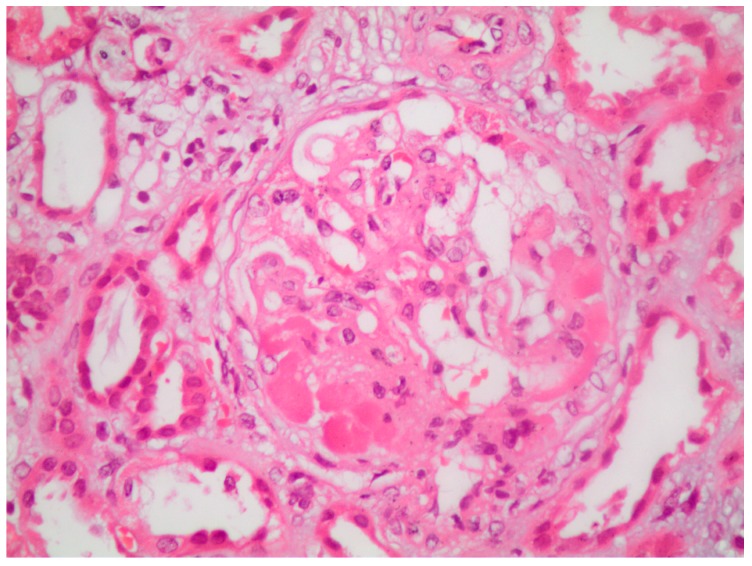
Segmental glomerular sclerosis with hyalinosis, as well as tubular dilation, tubular cell loss and detached epithelial cells into tubular lumens, in a patient with severe nephrotic syndrome due to focal segmental glomerulosclerosis (H&E X400).

Acute tubular necrosis in patients with NS is a sporadic but significant complication. It occurs due to hypovolemia in combination with hypoalbuminemia, often related to excessive diuresis. Hypovolemia may be the result of aggressive diuresis and the finding in physical examination of generalized edema. Undoubtedly, aggressive diuresis is necessary in cases with massive edema, especially if it is related with inability to walk, dyspnea, pleural effusions, and/or ascites. Interestingly, despite the marked expansion of the extracellular fluid volume, such patients may report symptoms of decreased effective circulating volume, including tachycardia, low blood pressure and oliguria. In most patients with NS and acute tubular necrosis, the pathophysiology of acute tubular injury and renal impairment are explained through the changed hemodynamics. Although the blood volumes may be preserved, the oncotic pressure remains significantly low in many patients with NS and thus, intravascular volume contraction is a key factor [32,57,58,59]. Other mechanisms which may cause renal dysfunction include low renal perfusion pressure, cast nephropathy, i.e., tubular obstruction with protein cast and interstitial edema [28,60]. In cases with minimal change disease and AKI it has been shown that the administration of albumin solutions inclined renal plasma flow although this finding has been disputed by others [25,61,62]. Furthermore, reductions of the epithelial slit pore length may be critical for the glomerular capillary permeability to water and small solutes while, in these patients, acute tubular injury is manifested by the dilation of tubular lumens with flattening of epithelium, sloughing of epithelial cells into tubular lumens, and interstitial edema with a few scattered inflammatory cells. Exposure to non-steroidal anti-inflammatory drugs (NSAIDS) also contribute to the development of glomerular lesions in minimal change disease. Diffuse foot process effacement of podocytes is a typical finding with the absence of brush border and basolateral infoldings in the proximal tubules and negative immunofluorescence or scattered IgM mesangial staining [63,64,65] (Figure 1).

#### Contrast Nephropathy

Nephrotic patients may also develop AKI after the administration of iodinated contrast media-enhanced radiographic procedures. The precise mechanism of acute tubular necrosis in cases with contrast nephropathy remains not well understood. Renal vasoconstriction and thus hypoxia in the renal tissue due to impaired viscosity, as well as a straight cytotoxic consequence of the contrast itself on the tubular cells [63,64,65] are considered as the primary factors. It is also unclear why the duration of acute tubular necrosis is shorter in cases with contrast induced nephropathy compared to acute tubular necrosis from other causes. It has been postulated that acute renal dysfunction comes as a result of functional changes rather than necrosis and thus, recovery occurs quickly. Tao et al., in studying a large cohort of matched patients, found that the risk of AKI, after the administration of intravenous contrast during computed tomography, was not higher in nephrotic patients [66]. Diagnosis of AKI in such cases is based upon the characteristic rise decrease in GFR within 24–48 h after the initiation of the contrast agent. Exclusion of other causes of AKI, using microscopic urinalysis and rarely a renal biopsy, is always required, while it should be differentiated from thrombotic complications of NS. Despite the fact that it is a reversible condition, it may occasionally be related to significant morbidity.

### 5.2. Acute Interstitial Nephritis

Acute interstitial nephritis is another setting of AKI, which is clinically characterized by the unexpected decline in renal function in combination with inflammation and edema of the renal interstitium. Suspicion and/or histopathologic documentation of acute interstitial nephritis in patients with NS should lead to a careful search for an etiologic agent, including infections, previous or concurrent administration of medicines such as antibiotics, NSAIDS, immune or neoplastic disorders. The most common antibiotics which may cause acute interstitial nephritis include cephalosporins, ciprofloxacin, ethambutol, isoniazid, macrolides, penicillin, rifampin, sulfonamides, tetracycline and vancomycin. Histologically, interstitial nephritis associated with NSAIDS is characterized by diffuse inflammatory infiltrates in the interstitium. The clinical picture typically includes renal insufficiency, low grade fever, rash and arthralgias. Often, some of the clinical sighs/symptoms are not present. NSAIDS have been known for their nephrotoxic potentials including their correlation with minimal change disease and with interstitial nephritis. They act through the inhibition of cyclooxygenase, a prostaglandin synthase, which is involved in the transformation of arachidonic acid to prostaglandins, prostacyclin, and thromboxanes [67,68]. More recent agents, such as selective cyclooxygenase-2 inhibitors, have anti-inflammatory and analgesic effects similar to classic NSAIDs with fewer adverse events. However, the risk of AKI appears not different to the use of different NSAIDs [69]. Risk factors for AKI induced by NSAIDs include history of chronic kidney disease, age, volume depletion and decreased effective arterial volume of any cause, other medications, i.e., diuretics and angiotensin-converting enzyme inhibitors or angiotensin receptor blockers, dosage and duration of therapy with NSAIDS [70].

### 5.3. Renal Vein Thrombosis

Thromboembolic events and renal vein thrombosis represent one of the most serious complications of NS and thus, AKI may occur, especially in cases with bilateral thrombosis. Estimates of the frequency of renal vein thrombosis among nephrotic patients range from 5%–60%, with an overall incidence of 35% [71]. This wide-ranging variability in the frequency of thrombotic events in NS is probably related to the characteristics of the patients included in the studies, i.e., symptomatic or clinically silent thrombotic events, degree of proteinuria, as well as the methods used for detection of thrombosis. In studies of consecutive or unselected patients without membranous nephropathy, who underwent venography, the prevalence of renal vein thrombosis ranges from 10% to 50% [72]. In a prospective study of 151 nephrotic patients, renal vein thrombosis was diagnosed in 22% of them, while one third of them had membranous nephropathy [73]. Overall, patients with membranous nephropathy and NS appear to be in a higher risk for thrombosis, when compared with nephrotic patients with other glomerular diseases [72,73,74,75,76,77,78,79]. In this regard, in a large cohort of 1313 patients with primary glomerular diseases, it was shown that the incidence of venous thromboembolic events was much higher among patients with membranous nephropathy (7.9% vs. 3% and 0.4% respectively). As a result, the histopathologic diagnosis was shown to be predictive for thrombosis occurrence after controlling for the 24 h proteinuria and serum albumin levels [74]. The risk of thrombosis has also been found to increase by the severity of hypoalbuminemia, a phenomenon, which is significant in patients with membranous nephropathy, as shown in a cohort of 898 patients with biopsy-proven disease [75]. The precise pathogenic mechanism is not known. The hypercoagulability disturbances and decreased fibrinolysis have been noted to be greater in patients with membranous glomerulopathy vs. those with other types of lesions [80,81]. In addition, the reduction in plasma volume may be more significant in membranous nephropathy [71]. A study which studied nephrotic patients specifically, who carried the diagnosis of membranous nephropathy and also experienced renal vein thrombosis, found immune complexes in their circulation which were not found in patients with membranous nephropathy without renal vein thrombosis [82]. It is speculated that these complexes may trigger the activation of the coagulation process directly or may activate factor XII [83]. Clinically, renal vein thrombosis may follow different scenarios, including abrupt onset of pain, renal dysfunction and macroscopic hematuria [84], although it may also quite often be silent [85]. In a prospective design, patients with renal vein thrombosis in the context of NS were noted to have two distinct types of clinical presentation; acute and chronic [73]. The setting of acute renal vein thrombosis includes a history of acute flank pain, hematuria, abnormal intravenous venogram and decline of GFR. Chronic renal vein thrombosis is more often asymptomatic. The significant incidence of renal vein thrombosis in nephrotic patients and especially with membranous nephropathy, together with the lack of symptoms, which is common, raises the question of how to handle these patients diagnostically, given the absence of information in asymptomatic patients and the fact that renal venography is an invasive procedure, not free of complications [71]. The use of ultrasonography, spiral computerized tomography with contrast and images by magnetic tomography may be the first step of the workup, as non-invasive procedures. However, the gold standard diagnostic test remains selective renal venography and thus, if any of the above reveals a high suspicion of thrombosis, then a renal venogram should be performed. Routine screening for renal vein thrombosis is not suggested for patients with NS, but selective high-risk patients, i.e., who have full blown NS with severe hypoalbuminemia (i.e., below 2 g/day) with symptoms and/or renal dysfunction should be investigated [86]. If a nephrotic patient experiences signs or symptoms of a renal infract accompanied by acute renal failure, then acute complete renal vein thrombosis is suspected and thus, a selective renal venogram should be performed, which might be combined with a simultaneous therapeutic procedure. Renal biopsy is of great importance to determine the cause of renal vein thrombosis, if not known. Membranous nephropathy, membranoprolifetive glomerulonephritis and minimal change disease are the leading causes, although any other diseases with NS can also present with renal vein thrombosis. In some instances, fibrin thrombi in blood vessels or glomerular capillaries may be seen while prominent congestion of glomerular or interstitial capillaries and a disproportionate degree of interstitial edema should always raise the possibility of renal vein thrombosis. Patients who receive a diagnosis of renal vein thrombosis, should be started on treatment with anticoagulatives immediately. Patients with symptomatic renal vein thrombosis are treated with unfractionated or low molecular weight heparin and then warfarin with a goal international normalized ratio of 2–3 [87,88,89,90]. Occasional patients with NS appear somewhat unaffected by heparin therapy, a fact which is related to severe anti-thrombin deficiency. The use of oral and parenteral direct thrombin inhibitors and factor Xa inhibitors have not been studied in patients with NS or impaired renal function, since such patients have been excluded from the related trials. Therapy with warfarin is given for a minimum of 6–12 months and should be continued as long as the patient remains nephrotic [71,86]. The use of oral and parenteral direct thrombin inhibitors and factor Xa inhibitors have not been studied in patients [91] with NS while patients with impaired renal function have been excluded from the studies of these agents. Systemic fibrinolytic therapy is not recommended in patients with AKI due to renal vein thrombosis, since it carries significant risk for bleeding, especially intracranial [92,93,94]. Anticoagulation for asymptomatic renal vein thrombosis may be identified through screening at the time of diagnosis of NS, which is not recommended without any clinical and/or laboratory indication or in case of other imaging studies. Besides, there is no data regarding the role of anticoagulation in patients with NS with asymptomatic renal vein thrombosis except from limited data reported in case series [73,88]. The need of prophylactic anticoagulation in cases with NS depends upon the incidence of thrombotic events in the patients, the effectiveness of anticoagulative therapy to prevent them and the associated risk of bleeding with this kind of treatment. In this regard, the data from prospective or controlled studies, comparing the risks associated with undiagnosed venous thrombosis with the risk of long-term anticoagulation, are limited. However, we estimated [95] the frequency of venous thromboembolic events in an inception cohort of 898 patients with primary membranous nephropathy versus the risk of bleeding using data from a systematic review of the literature. The probability of benefiting from a prophylactic regimen was evaluated by the risk of bleeding and the level of serum albumin. Patients who were scored with a high risk of bleeding, were not able to receive prophylactic anticoagulation and benefit of it irrespective of the level of serum albumin. In light of clinical practice and in order to assist the decision for prophylactic coagulation initiation in these patients, a tool was created to estimate the probability of benefit based on an individual’s bleeding risk, serum albumin level, and benefit-to-risk ratio (http://www.gntools.com) [95].

### 5.4. Warfarin (Anticoagulant) Associated Nephropathy

Another type of kidney injury, which comes as a result of overdosed anticoagulative therapy, has been described as anticoagulant related nephropathy. It represents a type of AKI which is a consequence of warfarin overdose [96,97]. Histologically it is characterized by acute tubular lesions and obstructed tubules with red blood cells casts [98]. This finding was documented in a series of nine biopsy proven cases who developed unexplained AKI following warfarin overdose. The precise incidence of this disorder in unknown, but it has been estimated that 17% of patients with international normalized ratio above 3 and no underlying kidney disease may experience an elevation in serum creatinine [99,100]. However, no histological data were revealed in this study and other causes of AKI might have been involved. Reasonably, nephrologists are happy with the risk which comes with a kidney biopsy in patients who need anticoagulation. One concern is the risk of thrombosis during the period anticoagulation is held, and the other the risk of hemorrhage from the kidney biopsy site after the systemic anticoagulation is resumed. The main diagnostic criterion for anticoagulant related nephropathy is glomerular hemorrhage following excessive anticoagulation. Notably, patients with abnormal glomerular membranes have been shown to be more vulnerable to this condition [100,101]. Glomerular bleeding leads to the formation of red blood cells casts, which cause obstruction in the renal tubules. This is the most striking histologic feature, as shown from specimens obtained from patients [98] and animal models [102,103,104]. Yet, the percentage of completely obstructed tubules seen by microscopy cannot always explain the severe deterioration of GFR [100,101,104]. It is possible that the small kidney specimen taken by a biopsy may not be representative of the real lesion. Besides, it has been hypothesized that another type of tubular epithelial cell injury may be involved, similar to the one which has been observed in patients with immunoglobulin A nephropathy or paroxysmal nocturnal hemoglobinuria [105,106]. Oxidative activity of heme and iron may be implicated in this type of tubular injury [107] or the anticoagulative agent itself may be directly toxic to the renal epithelial cell [104]. The major risk factor for developing anticoagulant related nephropathy is coagulopathy from warfarin or other anticoagulants use, although it is probably a multifactorial condition, since factors other than the degree of coagulopathy may be present and play a role in the development of AKI [98], including chronic renal impairment, diabetes mellitus, heart failure, arterial hypertension and glomerular disorders [98]. The clinical presentation is characterized by hematuria and subsequent renal dysfunction which usually occurs within 8 weeks from warfarin initiation [98]. From observational studies and animal models it has been shown that a few days with warfarin induced coagulopathy is enough to cause anticoagulant related nephropathy. Gross hematuria is less common than microscopic hematuria [97,98]. Absence of hematuria has also been reported, which is explained by the hypothesis that hemorrhaging glomeruli are shut down by the red blood cells casts and AKI, by the time these patients seek medical attention.

### 5.5. Aggressive Disease

Of all primary glomerulopathies, only the collapsing variant of focal segmental glomerulosclerosis may follow a course of rapid progression due to the disease itself. Focal segmental glomerulosclerosis is manifested by segmental glomerular sclerosis, usually associated with hyalinosis. Four distinct variants have been described, with the collapsing variant to be more ominous. It is seen in human immunodeficiency virus (HIV) patients and in cases with idiopathic focal segmental glomerulosclerosis. It is characterized by hyperplastic and hypertrophied podocytes with vacuoles and periodic acid shiff positive cytoplasmic droplets in association with glomerular tuft collapse and thus, often is exhibiting severe acute tubular injury and deteriorating renal function. Focal or more commonly widespread changes in the tubules can also be seen. Tubules are dilated and filled with pale-staining luminal precipitate. It has been historically associated with a significant and quick decline of GFR [108,109,110]. In a study of 62 patients, with this type of lesion, the collapsing group comprised of more African Americans (82.8%) and more women [111]. Patients with collapsing focal segmental glomerulosclerosis presented with more severe renal dysfunction and lower serum albumin level [111,112]. Primary collapsing focal segmental glomerulosclerosis represents an active, aggressive form of podocyte injury. Thomas et al. used a scoring system to describe specific pathologic findings where patients with collapsing focal segmental glomerulosclerosis had the highest total injury score [113]. Collapsing focal segmental glomerulosclerosis is most often seen in adults, with more males are affected, although this is not different from patients with focal segmental glomerulosclerosis without the collapsing lesion [114]. Patients with collapsing focal segmental glomerulosclerosis experience a short course from onset of symptoms to biopsy [109]. At presentation, patients have significantly higher levels of protein excretion with many patients excreting more than 10 grams per day. In addition, the time from onset of symptoms to renal biopsy has been shown to be significantly shorter, suggesting that a more fulminant disease course has been preceded prior to kidney biopsy. Weiss et al. have reported on six African Americans with renal insufficiency, massive proteinuria and renal biopsy showing glomerular collapse [115]. Laurin et al. however [111], in a retrospective study of patients, who received a diagnosis of collapsing focal segmental glomerulosclerosis between 1989 and 2012, assessed the rates of treatment response after adjusting for baseline characteristics and the type of immunosuppressive therapy. According to their results, the collapsing variant was not associated with a significantly worse renal survival, and probably the most important factor was shown to be the prompt diagnosis and initiation of immunosuppressive therapy in these patients [111].

Nevertheless, treatment of these cases is a challenge for clinicians as there are no prospective controlled trials and thus any approach is based principally upon clinical experience. The reported number of HIV-negative patients with collapsing focal segmental glomerulosclerosis, who have undergone complete remission in response to therapy is relatively small. A course of oral glucocorticoids for six months may be given as first line therapy (prednisone 1 mg/kg BW every day or 120 mg every other day for the first two months), then subsequently tapered over a minimum period of 6 months. We also administer antimicrobial prophylaxis against *Pneumocystis* pneumonia for the duration of glucocorticoid treatment. In cases with significant amounts of proteinuria, severe hypoalbuminemia and conserved GFR, a calcineurin inhibitor is added to the glucocorticoid regimen to accelerate reversal of the hypoalbuminemia and proteinuria. Notably, a combined treatment with glucocorticoids and other immunosuppressive agents was not related with better outcomes than therapy with glucocorticoids alone. A study which was performed retrospectively, and included 275 patients with biopsy proven focal segmental glomerulosclerosis, treated with immunosuppressive regimens, showed that for the collapsing variant there was no difference in the adjusted risk of end-stage renal disease between treatment with calcineurin inhibitors (with or without glucocorticoids) compared to treatment with glucocorticoids alone [111]. Besides, regimens including cyclophosphamide or chlorambucil were associated with unsatisfactory effectiveness and significant side effects [116].

### 5.6. Crescentic Glomerulonephritis Superimposition

**Figure 2:**
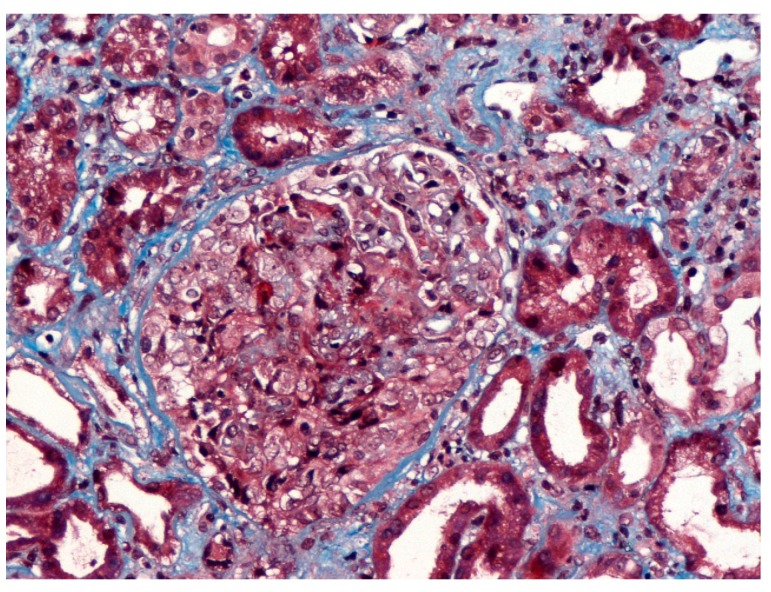
Cellular glomerular crescent in a patient with biopsy proven membranous nephropathy who developed rapidly progressive glomerulonephritis and acute kidney injury (AKI) associated with anti-neutrophil cytoplasmic antibodies (ANCA) antibodies (Masson X 400).

Occasionally, patients with NS may develop AKI as a result of disease transformation to rapidly progressive glomerulonephritis with crescent formation. This infrequent clinical scenario has been reported in patients with already diagnosed membranous nephropathy who presented with a rapid decline in renal function and switch from the nephrotic syndrome clinical setting to nephritic syndrome. In these cases, a new biopsy revealed that anti-glomerular basement membrane or anti-neutrophil cytoplasmic antibodies (ANCA) associated glomerulonephritis was superimposed on membranous nephropathy [117]. The first case of combined membranous and crescentic glomerulonephritis was reported by Klassen et al. [118] and was associated with anti-glomerular basement membrane antibodies. The author suggested that membranous nephropathy primarily damaged the glomerular basement membrane and released antigens that incited the anti-glomerular basement disease. Pettersson et al. reported a series of 17 patients with this kind of mixed lesion. Of those patients with follow up, 12 ended up in end stage renal disease, and the remaining responded to therapy with variable degrees of renal dysfunction [115]. Another mechanism of crescent formation in the context of membranous nephropathy has been associated with ANCA disease. In additional to other cases reported in the literature, Nasr et al. [119] described 14 cases with simultaneous diagnosis of ANCA-associated glomerulonephritis and membranous nephropathy. In 13 of them, patients with this mixed lesion was revealed at the first clinical presentation by histopathology while in 1 patient, membranous nephropathy preceded ANCA associated glomerulonephritis by 7 months. The majority of patients in this series presented with rapidly progressive renal dysfunction and nephrotic range proteinuria. The mean 24 h urine protein was 6.5 g, and 9 had full blown NS [119]. Thus, the development of crescentic glomerulonephritis in a background of a primary glomerulopathy should be included in the differential diagnosis of a patient with AKI. A second kidney biopsy is mandatory for these cases in order to explain AKI and make the final diagnosis. Besides, speedy diagnosis and therapy initiation is fundamental in all cases with rapidly progressive glomerulonephritis. Immunosuppressive therapy pertains to induction of remission with glucocorticoids and cyclophosphamide [120,121], given as intravenous pulses of methyl-prednisolone (7 mg/kg for 3 consecutive days) followed by oral prednisone (1 mg/kg for a minimum of 4 weeks), subsequently reduced over the next 3–5 months [122]. Cyclophosphamide is administrated, either intravenously as monthly pulses (0.5–1) g/m^2^ body surface are, or orally (2 mg/kg/day), adjusted by the leukocyte count. Additional interventions, such as plasma exchange [123] and/or administration of the anti-CD20 agent rituximab depends on the severity of renal impairment, the immunological environment surrounding this complication i.e., ANCA positive, anti-glomerular basement membrane antibody positive or pulmonary hemorrhage. Importantly, speed in diagnosis and early induction of immunosuppressive therapy are fundamental, because the type of lesion may follow a catastrophic course, leading to end stage renal histopathology within a few weeks (Figure 2).

### 5.7. Calcineurin Inhibitors Toxicity

Long term clinical experience in organ transplantation has shown that calcineurin inhibitors can induce renal dysfunction. This is a result of the varied bio-availabilities, targeted therapeutic range, and exhibition of a variety of adverse events depending on the patient and the serum titer of the drug. Patients with NS may need to be treated with calcineurin inhibitors, if they experience (i) several relapses while reducing the dose of glucocorticoids; (ii) resistance to treatment with glucocorticoids; (iii) significant toxicity related with glucocorticoids. Certain studies have showed that cyclosporine A is effective in the treatment of steroid-sensitive NS [124,125,126] with relapses after early withdrawal. Cyclosporine A is known to induce interstitial fibrosis [125] and vasoconstriction and subsequently decreased GFR [127,128]. The risk which is associated with these effects in case of long-term therapy with cyclosporine is a fact. However, usually it is a reversible effect resembling the clinical setting of prerenal disease [129]. The onset of acute calcineurin inhibitor nephrotoxicity may occur within hours to days, even years after the initiation of therapy. Factors predisposing to it include high dosing, older age, co-administration of NSAIDs, hypovolemia or excessive use of diuretics, exposure to medications which inhibit cytochrome P-450 3A4/5 (CYP3A4/5), thereby increasing exposure to calcineurin inhibitors metabolites, or drugs that inhibit P-glycoprotein-mediated efflux of calcineurin inhibitors from tubular epithelial cells, thereby increasing local renal exposure to calcineurin inhibitors. Also, genetic polymorphisms in the genes encoding CYP3A4/5 and P-glycoprotein have been identified to play a role in calcineurin inhibitor metabolism [130]. Patients with AKI related to calcineurin inhibitors toxicity should be checked for all the above risk factors in order to help quick recovery. While AKI from calcineurin inhibitors is a reversible condition following a prompt decrease in blood level, if it remains long-term it may turn to a chronic progressive renal disorder, which is a permanent condition [131,132]. Although most come from the use of cyclosporine in kidney transplant recipients, a similar pattern of renal injury has been documented for tacrolimus as well. A retrospective study in 16 children treated with calcineurin inhibitors for steroid resistant NS found that [133] AKI was very common (81.3%) counting for 0.7 AKI episodes per patient year of follow-up [133]. Yet, when the authors restricted the rate to patients who have achieved remission, the number was 0.34 episodes of AKI per patient year. Among those, 71% of AKI episodes recovered to the baseline serum creatinine value, while in 29% a new baseline serum creatinine was set. Another study of 119 children with idiopathic NS and AKI [10] reported drug toxicity as the second most frequent cause [134]. In contrast, [135] a retrospective study of 20 patients with steroid-resistant NS, who received cyclosporine A for 5 years, showed that, although there was an initial drop in GFR, renal function remained stable afterwards and no one developed chronic renal failure. Several studies with cyclosporine and tacrolimus in kidney transplantation demonstrated a relationship between whole blood or plasma cyclosporine concentrations and acute nephrotoxicity that was recovering with dose reduction [136,137]. Thus, patients with AKI attributed to calcineurin toxicity should be managed with close monitoring and diminishing of the daily dosage. Finally, identification of the genetic polymorphisms related with the drug-metabolizing enzymes might be useful in the assessment of individualized dosage algorithms for calcineurin inhibitors, as are substrates for CYP3A5 and P-glycoprotein [138], in order to avoid AKI renal dysfunction episodes.

## 6. Management of Patients with AKI in Patients with NS

General measures for the management of AKI include fluid management in order to restore extracellular volume and increase the effective circulating volume. The initial approach however is to assess the volume of the patient and determine if he is euvolemic, hypervolemic or hypovolemic. This assessment, which is based on accurate records of fluid input and output, daily body weights, physical examination, blood pressure and pulse measurements, will subsequently guide clinical practice in terms of fluid administration or not.

Replacement of albumin with or without diuretics in patients with NS has been a debate over the past decade. Nevertheless, long standing clinical experience has shown that treatment with diuretics as well as albumin administration results in diuresis and natriuresis in patients with edema and hypoalbuminemia. However, some patients do not respond to diuretics as expected, even with high doses of one drug or combined diuretic therapy. This phenomenon is probably due to the fact that the real intravascular compartment is relatively small while the neurohumoral systems, including the sympathetic nervous system, the antidiuretic hormone and renin-angiotensin II-aldosterone axons have been activated. In this regard, it has been shown that albumin infusion [139] increases the intravascular volume by withholding movement of liquids to the third space compartment. Yet, routine use of albumin solutions in NS with normal or increased plasma volume, is doubted. Furthermore, furosemide is highly bound to albumin (>90%), and consequently is not filtered at the glomerulus, while it is secreted in the proximal tubule. If the patient is severely hypoalbuminemic (<2 g/dL) [140], the proportion of the free drug which is available is bigger. Consequently, it diffuses into the tissues, with expansion in its volume of distribution, resulting in less delivery to the proximal tubule for secretion into the lumen. As a result, many clinicians end up combining loop diuretics with albumin to induce diuresis, natriuresis, and improve edema. The combination of furosemide and albumin is considered to restore, at least in part, the intravascular volume [141]. If furosemide is given in the same solution with albumin, it is considered even better for its secretion in the proximal tubule. However, even if furosemide is secreted in the proximal tubule, an amount of it is bound to the filtered albumin in the lumen. As a result, the free drug delivery to limb of Henle’s loop is further decreased, contributing to diuretic resistance. Hypertrophy of the distal tubular cells due to increased reabsorption of delivered NaCl have also been associated with resistance to diuretics [142]. Since the studies exploring the usefulness of combining furosemide and albumin to treat edema in patients with NS [142] are controversal, and the cost of albumin administration is too high, we suggest that the management of these patients should be individualized, and that combination therapy should be restricted to patients with resistance to therapy of more than one diuretic [142].

**Figure 3:**
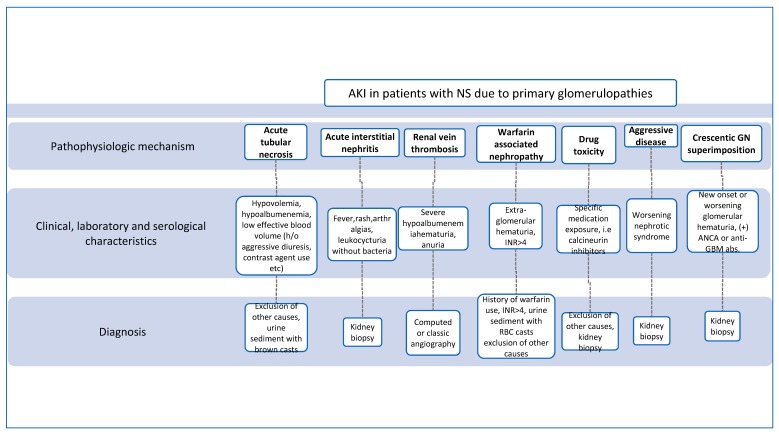
Algorithm for patients with AKI in the context of nephrotic syndrome (NS) due to primary glomerulopathies.

Serious and potentially life-threatening complications due to fluid overload, such as pulmonary edema, heart failure, hypertension, and being refractory to diuretic therapy, often require dehydration through dialysis sessions. In addition to hypervolemia, electrolyte abnormalities, metabolic acidosis and uremia may require renal replacement therapy temporarily, in cases with oligouria, especially in patients with acute tubular necrosis. In this setting, dialysis is continued until the regeneration of the tubular epithelium is evident. Pharmacologic management is also essential in these patients in order to avoid nephrotoxic medications and also adjust all renally excreted drugs to prevent the worsening of AKI. After the initial adjustment, which is made based on the calculated GFR the list of medications should be revisited regularly throughout the course of AKI and readjustments should be made as warranted if renal function improves or declines. In addition, levels should be routinely monitored for nephrotoxic medications with narrow therapeutic range such as aminoglycosides. Specific therapies depending on the etiology of AKI in patients with NS include anticoagulatives for renal vein thrombosis or immunosuppressive schemes for rapidly progressive glomerulonephritis as described above. In the latter case, early recognition and diagnosis by quick performance of kidney biopsy, is fundamental for its management with prompt initiation of immunosuppressive therapy (Figure 3).

Prophylactic measures for patients with NS in order to avoid AKI are generally limited and include the prevention of extreme restrictions in hydration or aggressive diuresis if not necessary, prophylactic anticoagulation, which may be given individually [95], according to the severity of NS, the specific features of each patient including age, coagulative disorders, hematocrit and propensity for hemorrhage and avoidance of nephrotoxic medications. Albumin replacement may be required in certain circumstances to avoid unwarranted intravascular hypovolemia.

## References

[1] Chertow G.M., Burdick E., Honour M., Bonventre J.V., Bates D.W. (2005). Acute kidney injury, mortality, length of stay, and costs in hospitalized patients. J. Am. Soc. Nephrol..

[2] Mehta R.L., Cerdá J., Burdmann E.A., Tonelli M., García-García G., Jha V., Susantitaphong P., Rocco M., Vanholder R., Sever M.S. (2015). International Society of Nephrology’s 0 by25 initiative for acute kidney injury (zero preventable deaths by 2025): A human rights case for nephrology. Lancet.

[3] Susantitaphong P., Cruz D.N., Cerda J., Abulfaraj M., Alqahtani F., Koulouridis I., Jaber B.L., Acute Kidney Injury Advisory Group of the American Society of Nephrology (2013). World incidence of AKI: A meta-analysis. Clin. J. Am. Soc. Nephrol..

[4] Hoste E.A., Schurgers M. (2008). Epidemiology of acute kidney injury: How big is the problem?. Crit. Care Med..

[5] Heung M., Steffick D.E., Zivin K., Gillespie B.W., Banerjee T., Hsu C.Y., Powe N.R., Pavkov M.E., Williams D.E., Saran R. (2016). Acute kidney injury recovery pattern and subsequent risk of CKD: An analysis of veterans health administration data. Am. J. Kidney Dis..

[6] Thakar C.V., Christianson A., Himmelfarb J., Leonard A.C. (2011). Acute kidney injury episodes and chronic kidney disease risk in diabetes mellitus. Clin. J. Am. Soc. Nephrol..

[7] Chawla L.S., Amdur R.L., Amodeo S., Kimmel P.L., Palant C.E. (2011). The severity of acute kidney injury predicts progression to chronic kidney disease. Kidney Int..

[8] Triverio P.A., Martin P.Y., Romand J., Pugin J., Perneger T., Saudan P. (2009). Long-term prognosis after acute kidney injury requiring renal replacement therapy. Nephrol. Dial. Transplant..

[9] Palevsky P.M., Liu K.D., Brophy P.D., Chawla L.S., Parikh C.R., Thakar C.V., Tolwani A.J., Waikar S.S., Weisbord S.D. (2013). KDOQI US commentary on the 2012 KDIGO clinical practice guideline for acute kidney injury. Am. J. Kidney Dis..

[10] Bellomo R., Ronco C., Kellum J.A., Mehta R.L., Palevsky P., Acute Dialysis Quality Initiative Workgroup (2004). Acute renal failure—Definition, outcome measures, animal models, fluid therapy and information technology needs: The Second international consensus conference of the acute dialysis quality initiative (ADQI) group. Crit Care.

[11] Mehta R.L., Kellum J.A., Shah S.V., Molitoris B.A., Ronco C., Warnock D.G., Levin A., Acute Kidney Injury Network (2007). Acute kidney injury network: Report of an initiative to improve outcomes in acute kidney injury. Crit Care.

[12] Levey A.S., Atkins R., Coresh J., Cohen E.P., Collins A.J., Eckardt K.U., Nahas M.E., Jaber B.L., Jadoul M., Levin A. (2007). Chronic kidney disease as a global public health problem: Approaches and initiatives—A position statement from Kidney Disease Improving Global Outcomes. Kidney Int..

[13] Molitoris B.A., Levin A., Warnock D.G., Joannidis M., Mehta R.L., Kellum J.A., Shah S.V., Molitoris B.A., Ronco C., Warnock D.G. (2007). Improving outcomes from acute kidney injury. J. Am. Soc. Nephrol..

[14] Fliser D., Laville M., Covic A., Fouque D., Vanholder R., Juillard L., Van Biesen W., Ad-hoc Working Group of ERBP (2012). A European Renal Best Practice (ERBP) position statement on the Kidney Disease Improving Global Outcomes (KDIGO) clinical practice guidelines on acute kidney injury: Part 1: Definitions, conservative management and contrast-induced nephropathy. Nephrol. Dial. Transplant..

[15] Leung K.C., Tonelli M., James M.T. (2013). Chronic kidney disease following acute kidney injury-risk and outcomes. Nat. Rev. Nephrol..

[16] Barasch J., Zager R., Bonventre J.V. (2017). Acute kidney injury: A problem of definition. Lancet.

[17] Cruz D.N., Bolgan I., Perazella M.A., Bonello M., de Cal M., Corradi V., Polanco N., Ocampo C., Nalesso F., Piccinni P. (2007). North East Italian Prospective Hospital Renal Outcome Survey on Acute Kidney Injury (NEiPHROS-AKI) Investigators. North East Italian prospective hospital renal outcome survey on acute kidney injury (NEiPHROS-AKI): Targeting the problem with the RIFLE Criteria. Clin. J. Am. Soc. Nephrol..

[18] Petäjä L., Røsjø H., Mildh L., Suojaranta-Ylinen R., Kaukonen K.M., Jokinen J.J., Salmenperä M., Hagve T.-A., Omland T., Pettilä V. (2016). Predictive value of high-sensitivity troponin T in addition to EuroSCORE II in cardiac surgery. Interact. Cardiovasc. Thorac. Surg..

[19] Qin J.P., Yu X.Y., Qian C.Y., Li S.S., Qin T.H., Chen E.Z., Lin J.D., Ai Y.H., Wu D.W., Liu D.X. (2016). Value of kidney disease improving global outcomes urine output criteria in critically III patients: A secondary analysis of a multicenter prospective cohort study. Chin. Med. J..

[20] Kellum J.A. (2015). Are outcomes from severe acute kidney injury really improving?. Am. J. Respir. Crit. Care Med..

[21] Hoste E.A., Bagshaw S.M., Bellomo R., Cely C.M., Colman R., Cruz D.N., Edipidis K., Forni L.G., Gomersall C.D., Govil D. (2015). Epidemiology of acute kidney injury in critically ill patients: The multinational AKI-EPI study. Intensive Care Med..

[22] Hsu R.K., McCulloch C.E., Ku E., Dudley R.A., Hsu C.Y. (2013). Regional variation in the incidence of dialysis-requiring AKI in the United States. Clin. J. Am. Soc. Nephrol..

[23] Nolasco F., Cameron J.S., Heywood E.F., Hicks J., Ogg C., Williams D.G. (1986). Adult-onset minimal change nephrotic syndrome: A long-term follow-up. Kidney Int..

[24] Hopper J., Ryan P., Lee J.C., Rosenau W. (1970). Lipoid nephrosis in 31 adult patients: Renal biopsy study by light, electron, and fluorescence microscopy with experience in treatment. Medicine.

[25] White R.H., Glasgow E.F., Mills R.J. (1970). Clinicopathological study of nephrotic syndrome in childhood. Lancet.

[26] Habib R., Kleinknecht C. (1971). The primary nephrotic syndrome of childhood. Classification and clinicopathologic study of 406 cases. Pathol. Annu..

[27] Bohlin A.B. (1984). Clinical course and renal function in minimal change nephrotic syndrome. Acta Paediatr. Scand..

[28] Smith J.D., Hayslett J.P. (1992). Reversible renal failure in the nephrotic syndrome. Am. J. Kidney Dis..

[29] Collister D., Pannu N., Ye F., James M., Hemmelgarn B., Chui B., Manns B., Klarenbach S., Alberta Kidney Disease Network (2017). Health care costs associated with AKI. Clin. J. Am. Soc. Nephrol..

[30] Rheault M.N., Zhang L., Selewski D.T., Kallash M., Tran C.L., Seamon M., Katsoufis C., Ashoor I., Hernandez J., Supe-Markovina K. (2015). AKI in children hospitalized with nephrotic syndrome. Clin. J. Am. Soc. Nephrol..

[31] Waldman M., Crew R.J., Valeri A., Busch J., Stokes B., Markowitz G., D’Agati V., Appel G. (2007). Adult minimal-change disease: Clinical characteristics, treatment, and outcomes. Clin. J. Am. Soc. Nephrol..

[32] Chen C.L., Fang H.C., Chou K.J., Lee J.C., Lee P.T., Chung H.M., Wang J.S. (2005). Increased endothelin 1 expression in adult-onset minimal change nephropathy with acute renal failure. Am. J. Kidney Dis..

[33] Keskar V., Jamale T.E., Kulkarni M.J., Kiggal Jagadish P., Fernandes G., Hase N. (2013). Minimal-change disease in adolescents and adults: Epidemiology and therapeutic response. Clin. Kidney J..

[34] Fenton A., Smith S.W., Hewins P. (2018). Adult minimal-change disease: Observational data from a UK centre on patient characteristics, therapies, and outcomes. BMC Nephrol..

[35] Meyrier A., Niaudet P. (2018). Acute kidney injury complicating nephrotic syndrome of minimal change disease. Kidney Int..

[36] Maas R.J., Deegens J.K., Beukhof J.R., Reichert L.J., Ten Dam M.A., Beutler J.J., van den Wall A.W.L., Rensma P.L., Konings C.J., Geerse D.A. (2017). The clinical course of minimal change nephrotic syndrome with onset in adulthood or late adolescence: A case series. Am. J. Kidney Dis..

[37] Moutzouris D.A., Herlitz L., Appel G.B., Markowitz G.S., Freudenthal B., Radhakrishnan J., D’Agati V.D. (2009). Renal biopsy in the very elderly. Clin. J. Am. Soc. Nephrol..

[38] Zhang Q., Zeng C., Cheng Z., Xie K., Zhang J., Liu Z. (2012). Primary focal segmental glomerulosclerosis in nephrotic patients: Common complications and risk factors. J. Nephrol..

[39] Earley L.E., Havel R.J., Hopper J., Grausz H. (1971). Nephrotic syndrome. Calif. Med..

[40] Bernard D.B. (1988). Extrarenal complications of the nephrotic syndrome. Kidney Int..

[41] Joven J., Villabona C., Vilella E., Masana L., Albertí R., Vallés M. (1990). Abnormalities of lipoprotein metabolism in patients with the nephrotic syndrome. N. Engl. J. Med..

[42] Kaysen G.A., de Sain-van der Velden M.G. (1999). New insights into lipid metabolism in the nephrotic syndrome. Kidney Int..

[43] Keane W.F. (1994). Effect of lipids on glomerular injury and progression of renal disease. Koninklijke Academie voor Geneeskunde van Belgie.

[44] Vaziri N.D. (2003). Molecular mechanisms of lipid disorders in nephrotic syndrome. Kidney Int..

[45] Shearer G.C., Stevenson F.T., Atkinson D.N., Jones H., Staprans I., Kaysen G.A. (2001). Hypoalbuminemia and proteinuria contribute separately to reduced lipoprotein catabolism in the nephrotic syndrome. Kidney Int..

[46] Rodríguez-Iturbe B., Herrera-Acosta J., Johnson R.J. (2002). Interstitial inflammation, sodium retention, and the pathogenesis of nephrotic edema: A unifying hypothesis. Kidney Int..

[47] Donckerwolcke R.A., Vande Walle J.G. (1997). Pathogenesis of edema formation in the nephrotic syndrome. Kidney Int. Suppl..

[48] Artunc F., Wörn M., Schork A., Bohnert B.N. (2019). Proteasuria—The impact of active urinary proteases on sodium retention in nephrotic syndrome. Acta Physiol..

[49] Jespersen B., Eiskjaer H., Mogensen C.E., Sørensen S.S., Pedersen E.B. (1995). Reduced natriuretic effect of atrial natriuretic peptide in nephrotic syndrome: A possible role of decreased cyclic guanosine monophosphate. Nephron.

[50] Klisic J., Zhang J., Nief V., Reyes L., Moe O.W., Ambühl P.M. (2003). Albumin regulates the Na^+^/H^+^ exchanger 3 in OKP cells. J. Am. Soc. Nephrol..

[51] Rostoker G., Behar A., Lagrue G. (2000). Vascular hyperpermeability in nephrotic edema. Nephron.

[52] Abrass C.K. (1997). Clinical spectrum and complications of the nephrotic syndrome. J. Investig. Med..

[53] Chuang C.H., Lee C.T., Cheng Y.F., Huang T.L., Hung K.H., Chen J.B. (2004). Bilateral renal infarctions and lower limbs artery thrombosis in a patient with nephrotic syndrome. J. Nephrol..

[54] Citak A., Emre S., Sâirin A., Bilge I., Nayir A. (2000). Hemostatic problems and thromboembolic complications in nephrotic children. Pediatr. Nephrol..

[55] Tesar V., Zima T., Kalousová M. (2003). Pathobiochemistry of nephrotic syndrome. Adv. Clin. Chem..

[56] Schlegel N. (1997). Thromboembolic risks and complications in nephrotic children. Semin. Thromb. Hemost..

[57] Kaysen G.A. (1994). Nonrenal complications of the nephrotic syndrome. Annu Rev. Med..

[58] Chamberlain M.J., Pringle A., Wrong O.M. (1966). Oliguric renal failure in the nephrotic syndrome. Q. J. Med..

[59] Shimoyama H., Nakajima M., Naka H., Maruhashi Y., Akazawa H., Ueda T., Nishiguchi M., Yamoto Y., Kamitsuji H., Yoshioka A. (2004). Up-regulation of interleukin-2 mRNA in children with idiopathic nephrotic syndrome. Pediatr. Nephrol..

[60] Koomans H.A. (2001). Pathophysiology of acute renal failure in idiopatic nephrotic syndrome. Nephrol. Dial. Transplant..

[61] Vande Walle J., Mauel R., Raes A., Vandekerckhove K., Donckerwolcke R. (2004). ARF in children with minimal change nephrotic syndrome may be related to functional changes of the glomerular basal membrane. Am. J. Kidney Dis..

[62] Eder H.A., Lauson H.D., Chinard F.P., Greif R.L., Cotzias G.C., van Slyke D.D. (1954). A study of the mechanisms of edema formation in patients with the nephrotic syndrome. J. Clin. Investig..

[63] Bohman S.O., Jaremko G., Bohlin A.B., Berg U. (1984). Foot process fusion and glomerular filtration rate in minimal change nephrotic syndrome. Kidney Int..

[64] Drumond M.C., Kristal B., Myers B.D., Deen W.M. (1994). Structural basis for reduced glomerular filtration capacity in nephrotic humans. J. Clin. Investig..

[65] Detrenis S., Meschi M., Musini S., Savazzi G. (2005). Lights and shadows on the pathogenesis of contrast-induced nephropathy: State of the art. Nephrol. Dial. Transplant..

[66] Tao S.M., Kong X., Schoepf U.J., Wichmann J.L., Shuler D.C., Zhou C.S., Lu G.M., Zhang L.J. (2018). Acute kidney injury in patients with nephrotic syndrome undergoing contrast-enhanced CT for suspected venous thromboembolism: A propensity score-matched retrospective cohort study. Eur. Radiol..

[67] Vane J.R. (1971). Inhibition of prostaglandin synthesis as a mechanism of action for aspirin-like drugs. Nat. New Biol..

[68] Brooks P.M., Day R.O. (1991). Nonsteroidal antiinflammatory drugs—Differences and similarities. N. Engl. J. Med..

[69] Schneider V., Lévesque L.E., Zhang B., Hutchinson T., Brophy J.M. (2006). Association of selective and conventional nonsteroidal antiinflammatory drugs with acute renal failure: A population-based, nested case-control analysis. Am. J. Epidemiol..

[70] Gooch K., Culleton B.F., Manns B.J., Zhang J., Alfonso H., Tonelli M., Frank C., Klarenbach S., Hemmelgarn B.R. (2007). NSAID use and progression of chronic kidney disease. Am. J. Med..

[71] Llach F. (1985). Hypercoagulability, renal vein thrombosis, and other thrombotic complications of nephrotic syndrome. Kidney Int..

[72] Singhal R., Brimble K.S. (2006). Thromboembolic complications in the nephrotic syndrome: Pathophysiology and clinical management. Thromb Res..

[73] Llach F., Papper S., Massry S.G. (1980). The clinical spectrum of renal vein thrombosis: Acute and chronic. Am. J. Med..

[74] Barbour S.J., Greenwald A., Djurdjev O., Levin A., Hladunewich M.A., Nachman P.H., Hogan S.L., Cattran D.C., Reich H.N. (2012). Disease-specific risk of venous thromboembolic events is increased in idiopathic glomerulonephritis. Kidney Int..

[75] Lionaki S., Derebail V.K., Hogan S.L., Barbour S., Lee T., Hladunewich M., Greenwald A., Hu Y., Jennette C.E., Jennette J.C. (2012). Venous thromboembolism in patients with membranous nephropathy. Clin. J. Am. Soc. Nephrol..

[76] Velasquez Forero F., Garcia Prugue N., Ruiz Morales N. (1988). Idiopathic nephrotic syndrome of the adult with asymptomatic thrombosis of the renal vein. Am. J. Nephrol..

[77] Liu Y.C., Wang H.Y., Pan J.S. (1989). Renal vein thrombosis in nephrotic syndrome—A prospective study of 54 cases. Zhonghua Nei Ke Za Zhi.

[78] Mahmoodi B.K., ten Kate M.K., Waanders F., Veeger N.J., Brouwer J.L., Vogt L., Navis G., van der Meer J. (2008). High absolute risks and predictors of venous and arterial thromboembolic events in patients with nephrotic syndrome: Results from a large retrospective cohort study. Circulation.

[79] Anderson F.A., Wheeler H.B., Goldberg R.J., Hosmer D.W., Patwardhan N.A., Jovanovic B., Forcier A., Dalen J.E. (1991). A population-based perspective of the hospital incidence and case-fatality rates of deep vein thrombosis and pulmonary embolism. The Worcester DVT study. Arch. Intern. Med..

[80] Kendall A.G., Lohmann R.C., Dossetor J.B. (1971). Nephrotic syndrome. A hypercoagulable state. Arch. Intern. Med..

[81] Wardle E.N., Menon I.S., Rastogi S.P. (1970). Study of proteins and fibrinolysis in patients with glomerulonephritis. Br. Med. J..

[82] Ooi B.S., Ooi Y.M., Pollak V.E. (1980). Identification of circulating immune complexes in a subpopulation of patients with membranous glomerulopathy. Clin. Immunol. Immunopathol..

[83] Berger J., Yaneva H. (1982). Hageman factor deposition in membranous glomerulopathy. Transpl. Proc..

[84] Harrison C.V., Milne M.D., Steiner R.E. (1956). Clinical aspects of renal vein thrombosis. Q. J. Med..

[85] Llach F., Koffler A., Massry S.G. (1977). Renal vein thrombosis and the nephrotic syndrome. Nephron.

[86] Rabelink T.J., Zwaginga J.J., Koomans H.A., Sixma J.J. (1994). Thrombosis and hemostasis in renal disease. Kidney Int..

[87] Kowal J., Figur A., Hitzig W.M. (1963). Renal vein thrombosis and the nephrotic syndrome with complete remission. J. Mt. Sinai Hosp. N. Y..

[88] Wagoner R.D., Stanson A.W., Holley K.E., Winter C.S. (1983). Renal vein thrombosis in idiopathic membranous glomerulopathy and nephrotic syndrome: Incidence and significance. Kidney Int..

[89] Glassock R.J. (2007). Prophylactic anticoagulation in nephrotic syndrome: A clinical conundrum. J. Am. Soc. Nephrol..

[90] Wu C.-H., Ko S.-F., Lee C.-H., Cheng B.-C., Hsu K.-T., Chen J.-B., Chien Y.-S., Yang C.-C., Huang M.-C., Chuang F.-R. (2006). Successful outpatient treatment of renal vein thrombosis by low-molecular weight heparins in 3 patients with nephrotic syndrome. Clin. Nephrol..

[91] Reynolds M.L., Nachman P.H., Mooberry M.J., Crona D.J., Derebail V.K. (2018). Recurrent venous thromboembolism in primary membranous nephropathy despite direct Xa inhibitor therapy. J. Nephrol..

[92] Markowitz G.S., Brignol F., Burns E.R., Koenigsberg M., Folkert V.W. (1995). Renal vein thrombosis treated with thrombolytic therapy: Case report and brief review. Am. J. Kidney Dis..

[93] Laville M., Aguilera D., Maillet P.J., Labeeuw M., Madonna O., Zech P. (1988). The prognosis of renal vein thrombosis: A re-evaluation of 27 cases. Nephrol. Dial. Transplant..

[94] Burrow C.R., Walker W.G., Bell W.R., Gatewood O.B. (1984). Streptokinase salvage of renal function after renal vein thrombosis. Ann. Intern. Med..

[95] Lee T., Biddle A.K., Lionaki S., Derebail V.K., Barbour S.J., Tannous S., Hladunewich M.A., Hu Y., Poulton C.J., Mahoney S.L. (2014). Personalized prophylactic anticoagulation decision analysis in patients with membranous nephropathy. Kidney Int..

[96] Ryan M., Ware K., Qamri Z., Satoskar A., Wu H., Nadasdy G., Rovin B., Hebert L., Nadasdy T., Brodsky S.V. (2014). Warfarin-related nephropathy is the tip of the iceberg:direct thrombin inhibitor dabigatran induces glomerular hemorrhage with acute kidney injury in rats. Nephrol. Dial. Transplant..

[97] Brodsky S.V., Nadasdy T., Rovin B.H., Satoskar A.A., Nadasdy G.M., Wu H.M., Bhatt U.Y., Hebert L.A. (2011). Warfarin-related nephropathy occurs in patients with and without chronic kidney disease and is associated with an increased mortality rate. Kidney Int..

[98] Brodsky S.V., Satoskar A., Chen J., Nadasdy G., Eagen J.W., Hamirani M., Hebert L., Calomeni E., Nadasdy T. (2009). Acute kidney injury during warfarin therapy associated with obstructive tubular red blood cell casts: A report of 9 cases. Am. J. Kidney Dis..

[99] An J.N., Ahn S.Y., Yoon C.H., Youn T.J., Han M.K., Kim S., Chin H.J., Na K.Y., Chae D.-W. (2013). The occurrence of warfarin-related nephropathy and effects on renal and patient outcomes in Korean patients. PLoS ONE.

[100] Kabir A., Nadasdy T., Nadasdy G., Hebert L.A. (2004). An unusual cause of gross hematuria and transient ARF in an SLE patient with warfarin coagulopathy. Am. J. Kidney Dis..

[101] Abt A.B., Carroll L.E., Mohler J.H. (2000). Thin basement membrane disease and acute renal failure secondary to gross hematuria and tubular necrosis. Am. J. Kidney Dis..

[102] Ware K., Brodsky P., Satoskar A.A., Nadasdy T., Nadasdy G., Wu H., Rovin B.H., Bhatt U., von Visger J., Hebert L.A. (2011). Warfarin-related nephropathy modeled by nephron reduction and excessive anticoagulation. J. Am. Soc. Nephrol..

[103] Ozcan A., Ware K., Calomeni E., Nadasdy T., Forbes R., Satoskar A.A., Nadasdy G., Rovin B.H., Hebert L.A., Brodsky S.V. (2012). 5/6 nephrectomy as a validated rat model mimicking human warfarin-related nephropathy. Am. J. Nephrol..

[104] Schützer K.M., Svensson M.K., Zetterstrand S., Eriksson U.G., Wåhlander K. (2010). Reversible elevations of serum creatinine levels but no effect on glomerular filtration during treatment with the direct thrombin inhibitor AZD0837. Eur. J. Clin. Pharmacol..

[105] Clarkson A.R., Seymour A.E., Thompson A.J., Haynes W.D., Chan Y.L., Jackson B. (1977). IgA nephropathy: A syndrome of uniform morphology, diverse clinical features and uncertain prognosis. Clin. Nephrol..

[106] Ballarín J., Arce Y., Torra Balcells R., Diaz Encarnación M., Manzarbeitia F., Ortiz A., Egido J., Moreno J.A. (2011). Acute renal failure associated to paroxysmal nocturnal haemoglobinuria leads to intratubular haemosiderin accumulation and CD163 expression. Nephrol. Dial. Transpl..

[107] Patel R.P., Svistunenko D.A., Darley-Usmar V.M., Symons M.C., Wilson M.T. (1996). Redox cycling of human methaemoglobin by H_2_O_2_ yields persistent ferryl iron and protein-based radicals. Free Radic. Res..

[108] D’Agati V.D., Kaskel F.J., Falk R.J. (2011). Focal segmental glomerulosclerosis. N. Engl. J. Med..

[109] Detwiler R.K., Falk R.J., Hogan S.L., Jennette J.C. (1994). Collapsing glomerulopathy: A clinically and pathologically distinct variant of focal segmental glomerulosclerosis. Kidney Int..

[110] Korbet S.M. (2012). Treatment of primary FSGS in adults. J. Am. Soc. Nephrol..

[111] Laurin L.P., Gasim A.M., Derebail V.K., McGregor J.G., Kidd J.M., Hogan S.L., Poulton C.J., Detwiler R.K., Jennette J.C., Falk R.J. (2016). Renal survival in patients with collapsing compared with not otherwise specified FSGS. Clin. J. Am. Soc. Nephrol..

[112] D’Agati V.D., Alster J.M., Jennette J.C., Thomas D.B., Pullman J., Savino D.A., Cohen A.H., Gipson D.S., Gassman J.J., Radeva M.K. (2013). Association of histologic variants in FSGS clinical trial with presenting features and outcomes. Clin. J. Am. Soc. Nephrol..

[113] Thomas D.B., Franceschini N., Hogan S.L., Ten Holder S., Jennette C.E., Falk R.J., Jennette J.C. (2006). Clinical and pathologic characteristics of focal segmental glomerulosclerosis pathologic variants. Kidney Int..

[114] Jennette J.C., Olson J.L., Schwartz M.M., Silva F.G. (2007). Hepinstall’s Pathology of the Kidney.

[115] Glicklich D., Haskell L., Senitzer D., Weiss R.A. (1988). Possible genetic predisposition to idiopathic focal segmental glomerulosclerosis. Am. J. Kidney Dis..

[116] Schwimmer J.A., Markowitz G.S., Valeri A., Appel G.B. (2003). Collapsing glomerulopathy. Semin. Nephrol..

[117] Pettersson E., Törnroth T., Miettinen A. (1984). Simultaneous anti-glomerular basement membrane and membranous glomerulonephritis: Case report and literature review. Clin. Immunol. Immunopathol..

[118] Klassen J., Elwood C., Grossberg A.L., Milgrom F., Montes M., Sepulveda M., Andres G.A. (1974). Evolution of membranous nephropathy into anti-glomerular-basement-membrane glomerulonephritis. N. Engl. J. Med..

[119] Nasr S.H., Said S.M., Valeri A.M., Stokes M.B., Masani N.N., D’Agati V.D., Markowitz G.S. (2009). Membranous glomerulonephritis with ANCA—Associated necrotizing and crescentic glomerulonephritis. Clin. J. Am. Soc. Nephrol..

[120] Falk R.J., Hogan S., Carey T.S., Jennette J.C. (1990). Clinical course of anti-neutrophil cytoplasmic autoantibody-associated glomerulonephritis and systemic vasculitis. The glomerular disease collaborative network. Ann. Intern. Med..

[121] de Groot K., Adu D., Savage C.O.S. (2001). The value of pulse cyclophosphamide in ANCA-associated vasculitis: Meta-analysis and critical review. Nephrol. Dial. Transpl..

[122] Couser W.G. (1988). Rapidly progressive glomerulonephritis: Classification, pathogenetic mechanisms, and therapy. Am. J. Kidney Dis..

[123] Jayne D.R., Gaskin G., Rasmussen N., Abramowicz D., Ferrario F., Guillevin L., Mirapeix E., Savage C.O.S., Sinico R.A., Stegeman C.A. (2007). Randomized trial of plasma exchange or high-dosage methylprednisolone as adjunctive therapy for severe renal vasculitis. J. Am. Soc. Nephrol..

[124] Hoyer P.F., Brodehl J., Ehrich J.H., Offner G. (1991). Practical aspects in the use of cyclosporin in paediatric nephrology. Pediatr. Nephrol..

[125] Tejani A., Lancman I., Pomrantz A., Khawar M., Chen C. (1988). Nephrotoxicity of cyclosporine A and cyclosporine G in a rat model. Transplantation.

[126] Hulton S.A., Jadresic L., Shah V., Trompeter R.S., Dillon M.J., Barratt T.M. (1994). Effect of cyclosporin A on glomerular filtration rate in children with minimal change nephrotic syndrome. Pediatr. Nephrol..

[127] Mihatsch M.J., Antonovych T., Bohman S.O., Habib R., Helmchen U., Noel L.H., Olsen S., Sibley R.K., Kemény E., Feutren G. (1994). Cyclosporin A nephropathy: Standardization of the evaluation of kidney biopsies. Clin. Nephrol..

[128] Mason J. (1990). Pharmacology of cyclosporine (sandimmune). VII. Pathophysiology and toxicology of cyclosporine in humans and animals. Pharmacol. Rev..

[129] Lamas S. (2005). Cellular mechanisms of vascular injury mediated by calcineurin inhibitors. Kidney Int..

[130] Kuehl P., Zhang J., Lin Y., Lamba J., Assem M., Schuetz J., Watkins P.B., Daly A., Wrighton S.A., Hall S.D. (2001). Sequence diversity in CYP3A promoters and characterization of the genetic basis of polymorphic CYP3A5 expression. Nat. Genet..

[131] Kahan B.D. (1989). Cyclosporine. N. Engl. J. Med..

[132] Kopp J.B., Klotman P.E. (1990). Cellular and molecular mechanisms of cyclosporin nephrotoxicity. J. Am. Soc. Nephrol..

[133] Beins N.T., Dell K.M. (2015). Long-term outcomes in children with steroid-resistant nephrotic syndrome treated with calcineurin inhibitors. Front. Pediatr..

[134] Yaseen A., Tresa V., Lanewala A.A., Hashmi S., Ali I., Khatri S., Mubarak M. (2017). Acute kidney injury in idiopathic nephrotic syndrome of childhood is a major risk factor for the development of chronic kidney disease. Ren. Fail..

[135] Kranz B., Vester U., Büscher R., Wingen A.M., Hoyer P.F. (2008). Cyclosporine-A-induced nephrotoxicity in children with minimal-change nephrotic syndrome: Long-term treatment up to 10 years. Pediatr. Nephrol..

[136] Henny F.C., Kleinbloesem C.H., Moolenaar A.J., Paul L.C., Breimer D.D., van Es L.A. (1985). Pharmacokinetics and nephrotoxicity of cyclosporine in renal transplant recipients. Transplantation.

[137] Laskow D.A., Vincenti F., Neylan J.F., Mendez R., Matas A.J. (1996). An open-label, concentration-ranging trial of FK506 in primary kidney transplantation: A report of the United States Multicenter FK506 Kidney Transplant Group. Transplantation.

[138] Fu R., Tajima S., Suetsugu K., Watanabe H., Egashira N., Masuda S. (2018). Biomarkers for individualized dosage adjustments in immunosuppressive therapy using calcineurin inhibitors after organ transplantation. Acta Pharmacol. Sin..

[139] Davison A.M., Lambie A.T., Verth A.H., Cash J.D. (1974). Salt-poor human albumin in management of nephrotic syndrome. Br. Med. J..

[140] Ponto L.L., Schoenwald R.D. (1990). Furosemide (frusemide). A pharmacokinetic/pharmacodynamic review (Part I). Clin. Pharmacokinet..

[141] Dorhout E.J., Roos J.C., Boer P., Yoe O.H., Simatupang T.A. (1979). Observations on edema formation in the nephrotic syndrome in adults with minimal lesions. Am. J. Med..

[142] Duffy M., Jain S., Harrell N., Kothari N., Reddi A.S. (2015). Albumin and furosemide combination for management of edema in nephrotic syndrome: A review of clinical studies. Cells.

